# Surgical Treatment of Non-embolized Patients with Nasoangiofibroma

**DOI:** 10.1016/S1808-8694(15)30607-8

**Published:** 2015-10-18

**Authors:** Adriano Santana Fonseca, Eriko Vinhaes, Viviane Boaventura, Nilvano Alves de Andrade, Lislane Andrade Dias, Vyrna Medeiros, Fernando Coifman

**Affiliations:** 1 Specialist in ENT and head and neck surgery by the HC/ UNICAMP. Specialist in Forensic Medicine at SSP/BA, Adjunct Professor of head and neck surgery at Santa Casa de Misericórdia da Bahia - Hospital Santa Izabel. Specialist in swallowing disorders and head and neck surgery at the Núcleo de Otorrinolaringologia e Estudos da Voz/Hospital da Bahia /Hospital Português.; 2 Specialist in ENT, otorhinolaryngologist, Adjunct Professor of rhinology at the ENT Department of the Santa Casa de Misericórdia da Bahia, Hospital Santa Izabel.; 3 MSc in pathology at FIOCRUZ/ UFBA, PhD student at FIOCRUZ UFBA, Adjunct Professor of ENT at Santa Casa de Misericórdia da Bahia, Hospital Santa Izabel.; 4 PhD in ENT at USP, Head of the ENT and Head and Neck Department at Santa Casa de Misericórdia da Bahia, Hospital Santa Izabel.; 5 MD, Resident at the ENT and Head and Neck Surgery Department at Santa Casa de Misericórdia da Bahia, Hospital Santa Izabel.; 6 MD, Resident at the ENT and Head and Neck Surgery Department at Santa Casa de Misericórdia da Bahia, Hospital Santa Izabel.; 7 MD, Resident at the ENT and Head and Neck Surgery Department at Santa Casa de Misericórdia da Bahia, Hospital Santa Izabel. Santa Casa de Misericórdia da Bahia, Hospital Santa Izabel.

**Keywords:** treatment, embolization, juvenile nasopharyngeal angiofibroma, skull base

## Abstract

Juvenile nasopharyngeal angiofibroma (JNA) is an uncommon tumor of the sphenopalatine foramen. Surgery combined with preoperative embolization has been the treatment of choice for JNA patients without intracranial invasion. This study aims to assess the viability of surgically treating non-embolized patients with JNA (types I-III according to Fisch).

**Materials And Method:**

This is a retrospective, descriptive study based on the medical records of 15 patients with histologically confirmed JNA (Fisch's types I- III), who underwent surgical treatment without pre-op embolization in our institution between 2000 and 2005.

**Results:**

Seven of the fifteen patients were approached endoscopically, four through the transantral approach, three were treated with the combined transmaxillary and endoscopic approach, and one with the combined transmaxillary and transpalatal approach. Six patients required intraoperatory blood transfusion, averaging volumes of 1.3 unit/patient. There were no cases of death or significant morbidity. Eleven of the fifteen patients were followed for an average of twelve months and 27% of them relapsed. Four patients did not comply with the follow-up scheme.

**Conclusion:**

Resection of JNF types I-III was safely completed in non-embolized patients. The observed levels of intraoperative bleeding, occurrence of complications, and rates of recurrence were close to those seen in embolized patients as found in the literature.

## INTRODUCTION

Juvenile nasopharyngeal angiofibroma (JNA) is an uncommon tumor that develops almost exclusively in young men. It accounts for 0.05% of all head and neck tumors^1^, ^2^. Although it is considered a benign neoplasm, this tumor is not encapsulated, may lead to local destruction and has high relapse rates1. This vascular tumor emerges mainly from the sphenopalatine foramen and may extend all the way to the middle cranial fossa^3^, ^4^.

According to the scheme proposed by Fisch^5^, these tumors may be contained within the nasal cavity and nasopharynx (I), invade the face sinuses or the pterygopalatine fossa (II), expand towards the infratemporal fossa, orbit, and parasellar region (III) or present intracranial invasion (IV).

Surgery is considered to be the best treatment option for JNA^6^ and many are the isolated and combined procedures and approaches to resect the tumor, such as the endoscopic, transmaxillary and transpalatine^7^. Intraoperative bleeding may occur in any of the approaches and has been one of the main perioperative complications. Preoperative arterial embolization has been used systematically to reduce the amount of bleeding and the use of blood byproducts during surgery with good results^4^, ^7^. However, there is no consensus as to the routine use of preoperative embolization, given its high cost and inconsistent availability of the procedure in care centers.

This paper aims to assess the possibilities of surgically treating patients for JNA without preoperative embolization looking at surgery time, volume of red cell packs, intra and postoperative complications, and tumor relapse rates.

## MATERIALS AND METHOD

This descriptive, retrospective study used data from the medical records of fifteen patients who underwent JNA resection surgery without preoperative tumor embolization between 2000 and 2005. All patients underwent nasofibroscopy with a flexible 3.2mm Machida scope and had sinus CT images taken.

Diagnostic criteria comprehended history of epistaxis and/or nasal obstruction, presence of angiomatous tumor under endoscopy, and CT image showing expansive formations in the nasal cavity with or without invasion into the sinuses, rhinopharynx, pterygomaxillary fossa, infratemporal fossa, orbit, and intracranial area, after contrast uptake. Tumors were rated based on the criteria developed by Fisch. Clinical diagnosis was confirmed postoperatively through histology exams done on the surgical specimens. All patients were operated on by the same surgeon.

None of the patients received adjuvant treatment such as hormone therapy, chemotherapy, or radiotherapy perioperatively.

Patients with previous history of JNA surgery were excluded. This study was approved by the Hospital Ethics and Research Committee under permit FR - 67401 and CAAE (CAAE - 0022.0.057.000-05).

Fentanyl (3Mcg/Kg), propofol (2.5mg/Kg), cisatracurium (0.15mg/Kg) were used in general anesthesia and dexamethasone (10mg) in anesthetic induction; and sevoflurane (2%) with remifentanil (0.2 to 0.5mg/Kg/min) in sustaining anesthesia. Blood pressure was kept at an average 60mmHg intraoperatively, and tranexamic acid (10mg/Kg) was administered as a prophylactic measure against bleeding. Volemic replacement was done with crystalloids and blood transfusion was indicated whenever hemoglobin was under 7g/dl.

When approaching patients endonasally, the surgeon used 0, 30 and 45 degree endoscopes (Comeg, Storz), nasal vasoconstriction with cotton swabs drenched in lidocaine and adrenalin solution (1:3000), and irrigation with saline solution at 0.9% and 4ºC. Patients were then submitted to a partial medial turbinectomy to expose the sphenoethmoidal recess and the sphenopalatine foramen region, defining the superior border of the tumor in the cases where it involved the tail or body of the concha. Sphenoidal sinusotomy was done via the sphenoethmoidal recess and ethmoidectomy with inferior and posterior traction of the tumor when the neoplasm involved the posterior-superior portion of the nasal fossa. Maxillary antrostomy, with detachment of the posterior fontanel to expose the sphenopalatine foramen, was indicated to determine the lateral borders of the tumor, in the pterygopalatine fossa or the infratemporal fossa. The tumor's arterial pedicles (mainly the often numerous terminal branches of the internal maxillary artery) that surround it inferiorly and superiorly were dissected and ligated with a monopolar electrocautery. A bipolar cautery was used in the vessels close to the meninges and the optic nerve, whereas neurosurgery 200 clips were placed in the larger diameter vessels.

In type-I patients, after vascular ligation, the tumor was pulled towards the rhinopharynx until it was completely detached. In type II and III patients, part of the palatine bone vertical lamina and of the maxillary sinus posterior wall were removed endoscopically, thus exposing the pterygopalatine fossa and part of the infratemporal fossa. The transeptal approach as described by Wormald8 was preferred in patients operated on more recently. Vascular ligation was conducted after the lateral borders of the tumor were identified as described above. The tumor was then detached up to its posterior borders, in the inferior orbital fissure and the venous pterygoid plexus.

When the lateral border could not be reached endoscopically, the transmaxillary approach was used in a medial labial rhinotomy or in a facial medial degloving procedure, as described by Denker and Caldwell-Luc respectively. The transpalatine approach with microscope assisted dissection combined with the transmaxillary approach was used in one case where a bulky tumor was adhered posteriorly.

Isolated transmaxillary approach was used in tumors invading the entire nasal fossa, regardless of type, that rendered impossible the use of the endoscopic approach.

All patients stayed in intensive care units for 24-48 hours after surgery.

Nasal packing, when required, was removed after 48-72 hours. Nasal irrigation with hypertonic saline solution was indicated after hospital discharge and follow-up was conducted for nasofibroscopy exams at the institution's outpatient ward. Endoscopic CT face scans were taken whenever relapsing tumors were suspected.

## RESULTS

All fifteen patients operated for JNA resection without embolization were staged based on the model proposed by Fisch. Eight patients (53%) had type II disease, followed by four (27%) with type III, and three (20%) with type I tumors. None of the patients had type IV disease. The endonasal and transmaxillary approaches were elected for patients with disease types I and II. Combined transmaxillary and endonasal or transmaxillary and transpalatine approaches were reserved for type III tumors.

Age averaged between 17 and 19 years within the three groups of patients. Surgery took slightly longer for type II tumors, but no statistically significant difference was found between the three groups. Average surgery length was 140 minutes. Six of the fifteen patients had intraoperative bleeding requiring blood transfusion. They averaged 1.3 units of red cell pack per patient.

Three patients had postoperative bleeding, two from group II and one from group III. Bleeding episodes were controlled by ligating the maxillary artery. All patients were discharged without requiring nasal packing. There were no postoperative complications.

All fifteen patients who underwent surgery were followed up for 12-63 months.

Three of the patients (27%) had relapsing tumors and were referred to tumor resection. None had relapsing tumors after the second procedure.

The data above is described on the chart below.

## DISCUSSION

Some authors believe embolization is indispensable when resecting nasopharyngeal angiofibromas endoscopically8,9. Most papers in the literature comparing surgical treatment with and without embolization have demonstrated reduced intraoperative blood loss and fewer blood transfusions when embolization is done^10^, ^12^. Blood loss is reduced by 836 to 1200 ml per non-embolized patient; and by 400 to 600 ml per embolized patient^10^, ^11^, ^12^, ^13^, ^14^, ^15^, ^16^, ^17^, ^18^, ^19^, ^20^, ^21^, ^22^, ^23^, ^24^, ^25^, ^26^.

**Chart:**
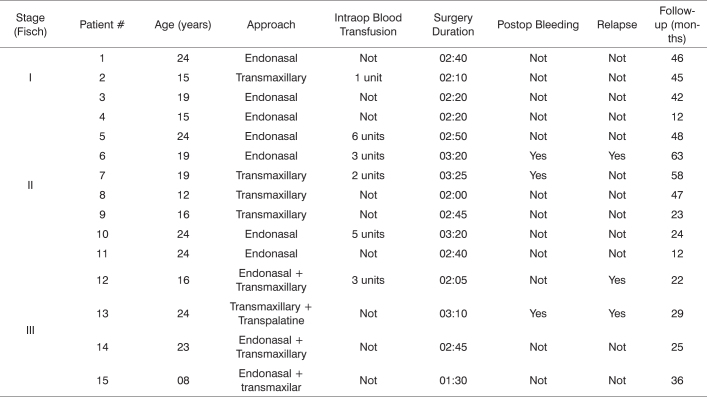

In terms of blood transfusion, the values ranged between 3 and 4.4 units of red cell pack per patient in the non-embolized group, when compared to 0 to 2 units in the group of embolized patients^11^, ^12^, ^13^, ^14^, ^15^, ^16^, ^17^, ^18^, ^19^, ^20^, ^21^, ^22^, ^23^, ^24^, ^25^, ^26^.

This study comprehended patients with disease types I to III according to Fisch. Intraoperative blood transfusion volumes were comparable to those found by other authors looking at preoperative embolization - 0 to 2 units per patient^11^, ^12^, ^13^, ^14^, ^15^, ^16^, ^17^, ^18^, ^19^, ^20^, ^21^, ^22^, ^23^, ^24^, ^25^, ^26^ against 1.3 units per patient in this study. Bleeding was reduced as a result of the adopted surgical tactic, which included systematic tumor vascular ligation in the beginning of the procedure, approaching not only the maxillary artery branch, but also all other arterial branches irrigating the neoplasm. By maintaining the patients’ blood pressure at a lower level (60mmHg) until the tumor has been removed and tranexamic acid applied, it is believed that intraoperative bleeding was significantly reduced.

Moulin et al. do not indicate embolization as a routine preoperative procedure. Considering the blood losses of embolized and non-embolized patients, the authors concluded that the procedure should only be offered to patients with larger tumors^12^. Petruson et al., looking at intraoperative bleeding, did not encounter statistically significant differences between embolized and non-embolized patients^13^. The authors found higher JNA relapse rates among embolized patients. Mc Combe et al. suggested that preoperative embolization may compromise the identification of surgical margins as it reduces tumor size, consequently increasing chances of relapse^14^.

Many papers also point out the benefits of preoperative arterial embolization in controlling intraoperative bleeding^8^, ^9^, ^15^, ^17^, reducing it by as much as fifty percent^15^, thus allowing the full excision of the tumor^16^, lower relapse rates and neurological complications^9^, ^17^, and reduced surgery duration when associated with the endoscopic approach alone^18^. Nevertheless, the most important prognostic factor for relapse is early staging and knowing the extension of the tumor^19^.

Maxillary artery embolization is a relatively safe invasive procedure. Complication rates may be as high as 27%20, most of which are temporary^21^ in spite of the few (2%) severe ones^22^. Such complications may include occlusion of the central retinal artery and consequent temporary blindness^23^, oronasal fistula due to tissue necrosis^24^, occlusion of the middle cerebral artery followed by stroke, and occlusion of the ophthalmic artery^25^.

Preoperative embolization may cost between USD 2,000 and USD 11,000^20^, ^21^,^27^, being therefore a procedure that most public and non-profit hospitals in Brazil cannot afford. We could not find indexed papers discussing the costs of this procedure in Brazil.

## CONCLUSION

This study presents a review of fifteen JNA patients who underwent surgery without preoperative embolization within a five-year span. Intraoperative and postoperative complication rates and blood loss levels were similar to those found in the literature for patients submitted to preoperative embolization, thus indicating that the procedure without preoperative embolization can be safely offered to selected patients. Additional comparative studies must be conducted so that a treatment routine can be developed for these patients.
